# Spinal arteriovenous malformation in a pediatric patient with a history of congenital syphilis: a case report

**DOI:** 10.1186/s12887-021-02707-y

**Published:** 2021-05-19

**Authors:** Mia J. Bertoli, Kruti Parikh, David Klyde, Catherine A. Mazzola, Shridevi Pandya Shah

**Affiliations:** 1 Rutgers New Jersey Medical School, Newark, NJ USA; 2 Department of Anesthesiology, Rutgers New Jersey Medical School, Newark, NJ USA; 3 Department of Radiology, Rutgers New Jersey Medical School, Newark, NJ USA; 4 Department of Neurosurgery, New Jersey Pediatric Neuroscience Institute, Morristown, NJ USA

**Keywords:** AVM embolization, SSEP monitoring, Evoked potentials, Spinal cord, Pseudoaneurysm, Congenital syphilis

## Abstract

**Background:**

Spinal arteriovenous malformations in children are extremely rare and pose great risk for intraoperative hemorrhage. Congenital syphilis sometimes presents with vascular symptoms, however, there is little published on patients with a history of congenital syphilis presenting with spinal arteriovenous malformations.

**Case presentation:**

A 15-month-old female with a history of congenital syphilis presented with urinary retention, fever, and subacute onset of paraplegia. MRI showed a lesion at T8-L1, angiogram was performed which confirmed the presence of a complex type IVc arteriovenous malformation and fistula from Artery of Adamkiewicz at L1-L2. It also showed peri medullary dilated veins and a pseudoaneurysm that compressed the spinal cord at T8-T10. Somatosensory evoked potentials and motor-evoked potentials were not recordable on the bilateral lower extremities prior to surgery. Once the patient was optimized for surgery, osteoplastic laminotomies from T6-T12 were performed. The dura was opened and the intradural, intramesenchymal hematoma was evacuated. There were two episodes of brisk arterial bleeding with hypotension during resection of the hematoma. The patient was taken to the angiography suite from the OR to successfully coil the large aneurysm. Intraoperative spinal cord monitoring remained undetectable in the bilateral lower extremities. The patient’s paraplegia remained unchanged from preoperative presentation.

**Conclusion:**

Congenital syphilis may present with vascular changes that might impact surgical approaches and treatment outcomes in patients with spinal arteriovenous malformations. Preparation for massive transfusion and intraoperative monitoring are imperative in ensuring a safe perioperative experience.

## Background

Arteriovenous malformations (AVMs) are abnormal connections between arteries and veins that have a high risk of rupture. Spinal AVMs occur in only about one-tenth of all central nervous system AVMs. Pediatric spinal AVMs are extremely rare and are usually only symptomatic during adulthood, therefore they are most diagnosed later in life. Males are more commonly affected than females, and in children, spinal AVMs are usually diagnosed after 10 years of age. The most common symptoms of spinal AVMs are back pain, impaired sensation, weakness, disturbed micturition and defecation, and subarachnoid hemorrhage [1].

Spinal AVMs are categorized as types I-IV that differ by where and when they present. Type I forms spontaneously in the dura during adulthood, while type II forms within the spinal cord itself. Type III forms during development in utero, and type IV forms in the pia mater of the spinal cord [2]. Since hemorrhage is the most common intraoperative complication, from the anesthesia standpoint, preparation for massive transfusion is imperative prior to and during surgery. We present a case of a 15-month-old female with a potentially difficult anesthetic due to spinal AVM and a history of congenital syphilis.

## Case presentation

**Fig. 1:**
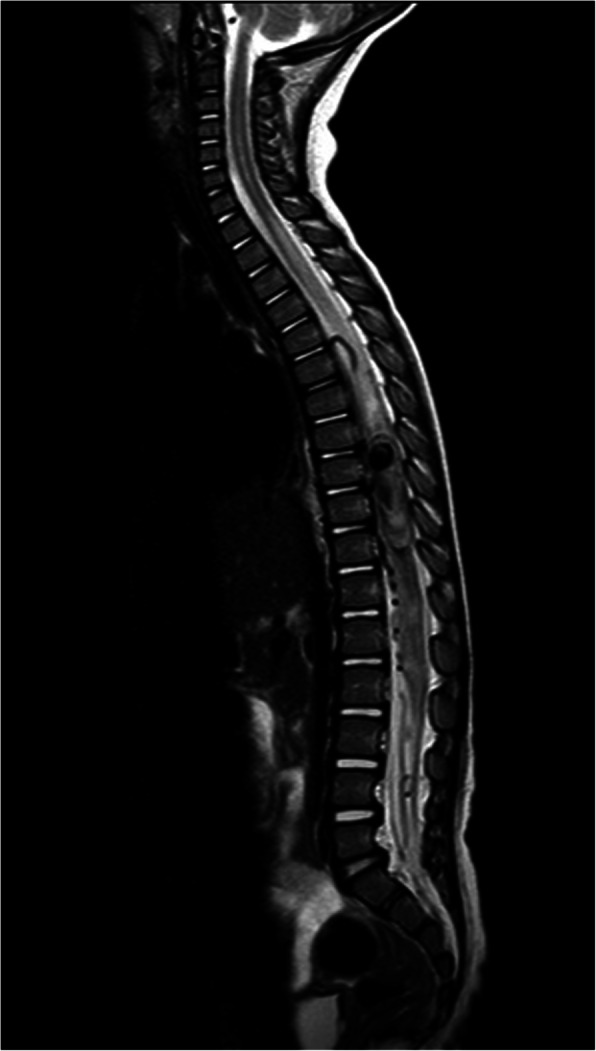
Sagittal T2 image of the spine demonstrates a mass with heterogeneity and flow voids from the level of T9 to T12 depicting the spinal AVM with pseudo aneurysm. Additionally, at the level of T6/T7 there is a flow void corresponding to the spinal artery of Adamkiewicz (anterior spinal artery) which demonstrates arteriomegaly

A 15-month-old 12.7 kg female presented with 1 week of fever, urinary retention, and subacute onset of paraplegia 1 day prior to admission. Her medical history was significant for birth at 36 weeks’ gestation and a 10-day hospitalization for treatment of congenital syphilis. Initial presentation was at another hospital where lumbar puncture showed leukocytosis, elevated protein, positive red blood cells, and no glucose. Blood and cerebral spinal fluid (CSF) cultures were negative, along with rapid CSF polymerase chain reaction. The patient was started on ceftriaxone, vancomycin, and acyclovir. MRI showed a lesion from T8-L1 (Fig. 1). The patient was transferred to our hospital for further management. She was able to move her upper but not her lower extremities at presentation to emergency room.

**Fig. 2:**
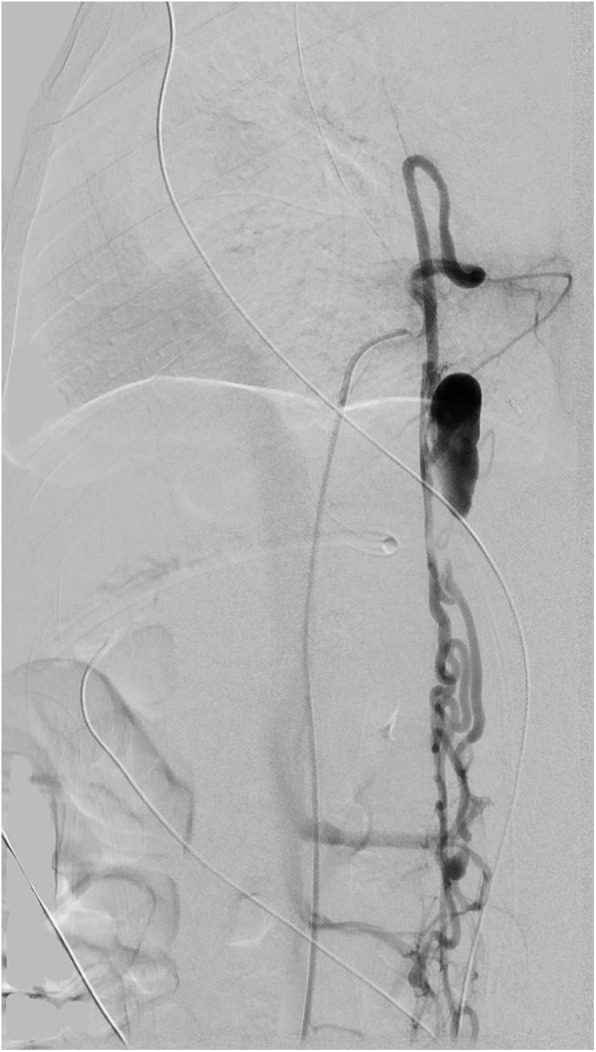
Digital subtraction angiogram in lateral projection of the injection of the anterior spinal artery at T8 with its hairpin turn feeding the AVM and the pseudoaneurysm which extends from T9 to T12. The venous plexus is contrast filled inferiorly with opacification of the draining veins and slight opacification of the IVC

**Fig. 3:**
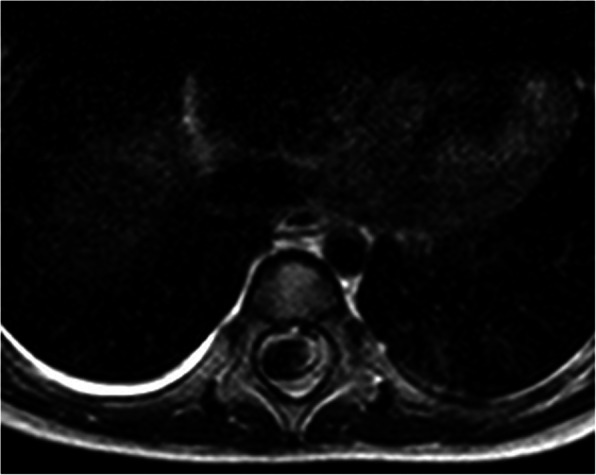
Axial T2 weighted image at T10 level showing the flow void of the AVM pseudoaneurysm occupying nearly the entire spinal canal and compressing the spinal cord from right to left

The emergent angiogram was performed under general anesthesia. Patient received combined intravenous and inhalation induction with propofol and sevoflurane. Two large bore IVs were established in anticipation of blood loss. The patient was found to have a complex type IVc AVM (Fig. 2) and fistula from the Artery of Adamkiewicz at L1-L2. She also had perimedullary dilated veins and a pseudoaneurysm that measured 2.5 cm × 1.5 cm × 1.5 cm. The pseudoaneurysm compressed the spinal cord at T8-T10 (Fig. 3), displacing it from right to left.

Interventional radiology felt at the time that the spinal AVM could not be embolized. The patient was transferred to the pediatric intensive care unit and was left intubated and sedated to reduce the risk of agitation induced rupture. Patient was optimized for surgical resection by raising hemoglobin to 14 g% and administering intravenous steroids. Two days later, she was brought to the operating room. The patient was prepared with establishment of central line, arterial line, and foley catheter. She was placed prone upon jelly roll bolsters below her sternum and iliac crests. Baseline spinal cord monitoring of somatosensory evoked potentials (SSEPs) and transcortical motor evoked potentials (MEPs) were clear only on the bilateral upper extremities. Bilateral lower extremities did not have recordable SSEPs or MEPs prior to surgery. Electromyography was quiet at baseline.

**Fig. 4:**
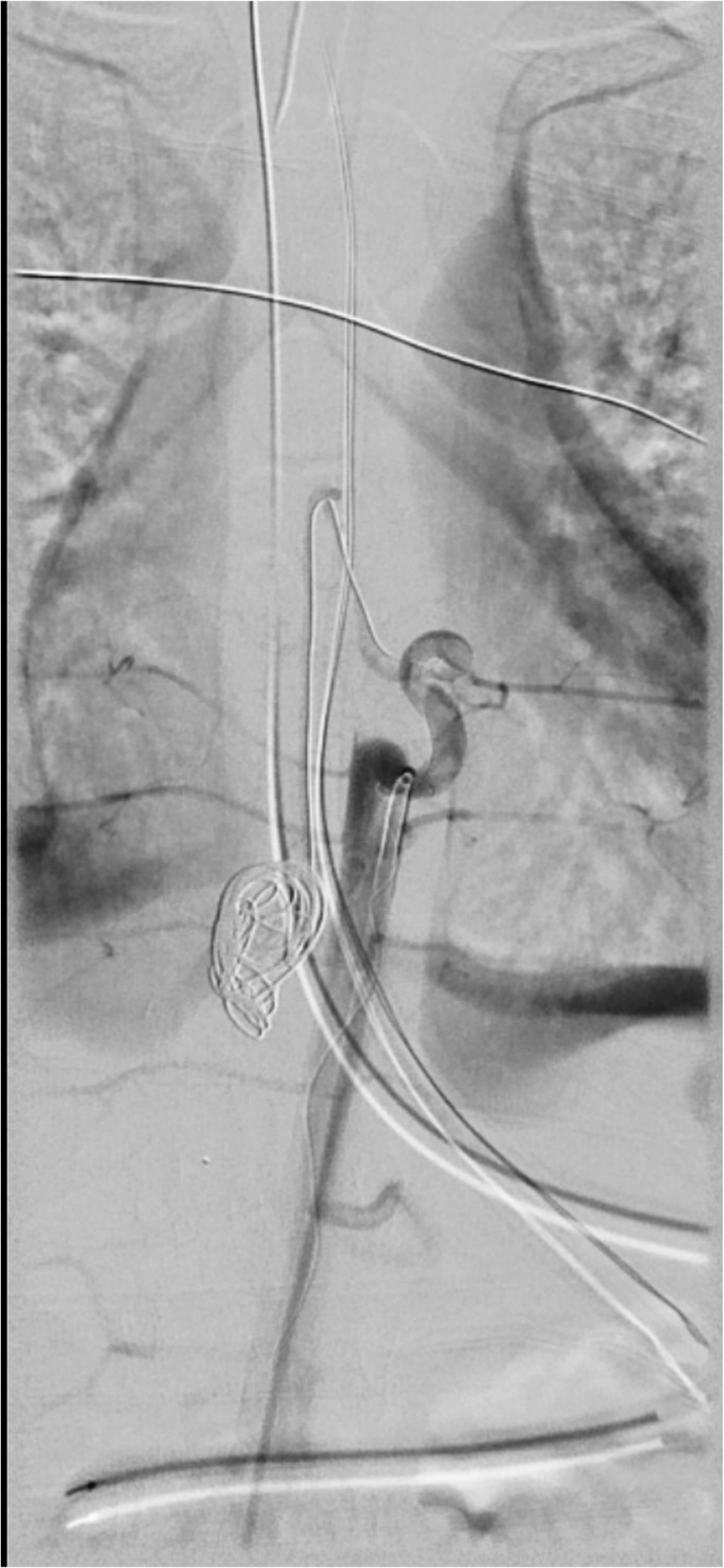
Digital subtraction angiogram of the origin of the anterior spinal artery at T8 on the left. The hairpin turns and the AVM pseudoaneurysm have been embolized with a detachable coil. There is no opacification of the spinal artery except for its origin. The pseudoaneurysm is occluded. Reflux into the intercostal arteries is demonstrated

Osteoplastic laminotomies from T6-T12 were performed. The dura was opened and the intradural, intramesenchymal hematoma was evacuated. There were two episodes of brisk arterial bleeding with hypotension during resection of the hematoma. She was transfused with 500 cc of packed cells, 100 cc of fresh frozen plasma, 120 ml of albumin, and 750 ml of crystalloids. Total estimated blood loss was 600 ml. Patient required boluses of epinephrine during episodes of bleeding. Infusions of pressor medications was not needed intraoperatively. Hemoglobin was maintained at or above 10 g/dl. Hemostasis was obtained with aneurysm clip placement after bipolar coagulation, cottonoid application, and gentle pressure. Once hemostasis was achieved, the surgery was stopped because the arterial supply from the ventral side of the aneurysm could not be surgically secured. The dura was left open secondary to spinal cord swelling and prominent aneurysm clips were added. The spinal cord was covered with an artificial dural substitute. The fascia and skin of the thoracolumbar spine were closed. Postoperatively, the patient was administered antibiotics and steroids. She was kept sedated and ventilated until the osteoplastic laminotomies were replaced and the dorsal elements of the spine were reconstructed. The patient was taken to the angiography suite from the OR to coil the large aneurysm (Fig. 4). A week later, the child was brought back to the OR for removal of aneurysm clips and reconstruction of posterior elements of the lamina. Intraoperative SSEPs and MEPs remained undetectable in lower extremities.

The patient remained paraplegic postoperatively. She received Botox injections to the gastrocnemius bilaterally and the left tibialis posterior about 6 months after surgery. The patient’s mother thought the Botox helped, and she is doing therapies with her at home but wishes to go back to formal therapy after COVID-19. Repeat spinal angiogram performed 1 year later showed no residual or recurrent arteriovenous malformation. Her current medications include baclofen 5 ml three times a day, sulfamethoxazole-trimethoprim 200–40 mg/5 ml oral suspension daily, Miralax and Dulcolax PRN. The patient is currently followed up by a urologist. She has some sensation in the lower legs but remains paraplegic with neurogenic bowel and bladder syndrome. She currently undergoes bladder catheterization every 3 h.

## Discussion and conclusion

Congenital syphilis cases have had a 46% increase in incidence in the United States since 2012. Untreated syphilis is a multisystem disease that can present with hematologic, neurologic, skeletal, oral, and cutaneous symptoms [3]. *Treponema pallidum* infection spreads via hematogenous dissemination that can result in inflammation of the vasa vasorum and granuloma formation in blood vessels. Syphilitic aneurysms are unlikely to rupture due to the granulomatous scarring of the intima media [4]. Syphilis can present with spinal and vascular symptoms that may result in secondary paraplegia. One of the first documented cases of paraplegia secondary to syphilis showed inflammation of the meninges and thickened and occluded vessels surrounding the spinal cord [5]. It is unclear if our patient developed vascular symptoms due to hematogenous spread of *T. pallidum* or if it was due to a congenital defect. In our opinion, our patients’ congenital syphilis was coincidental and unrelated to the spinal AVM. This assumption is since the child had otherwise normal development and good health since birth. There are no long-term sequelae of *T. pallidum* infection in our patient, such as rhagades, condyloma lata, Mulberry molars, saddle nose deformity, frontal bossing, or saber shins, to name a few. The patient was negative for Hutchinson’s triad, which is specific for congenital syphilis. Hutchinson’s triad includes Hutchinson’s teeth, interstitial keratitis, and cranial nerve eight deafness [6].

There are risks in using angiography in pediatric patients. This includes the need for sedation and radiation related hazards [7]. General anesthesia is usually needed when children are required to stay still for longer periods of time. One needs to look at the challenges associated with general anesthesia in this age group. Since children have smaller vessels, the concern of angiography is that the catheter can cause the vessels to dissect, spasm, or occlude to the point of ischemia [8]. Although there are risks associated with angiography in children, the benefits in our case were determined to outweigh the risks. Surgery was not possible without an angiogram. The nearby hospital our patient was being treated at did not have angiography equipment. The patient had to be transferred to our hospital to receive an accurate diagnosis and surgical intervention.

Spinal AVMs in the pediatric population are most frequently located in the thoracolumbar region. Our patient presented with a large AVM and fistula from the Artery of Adamkiewicz at T8 to L2. Hemorrhage is a common occurrence among patients diagnosed with spinal AVM, with most of the bleeds presenting in the subarachnoid space [1]. In our case, the patients’ pseudoaneurysm was compressing the spinal cord and was at a high risk for recurrent rupture. The decision was made to postpone surgical intervention in preparation for perioperative hemorrhage. This included hemoglobin correction and having blood products available. The pseudoaneurysm leaked after the dura was exposed which required aggressive resuscitation with blood products and pressors. Anesthetic management for spinal AVMs includes preparation for the hemodynamic changes that may occur perioperatively due to hemorrhage.

The anterior spinal artery (spinal artery of Adamkiewicz) supplies blood to the anterior horn and anterior portion of the lateral columns at each level from T8 to the conus medullaris region. Traumatic or ischemic injury to this artery will cause anterior spinal artery syndrome. Symptoms of onset may be rapid, from 2 min to several hours. Symptoms include intractable back pain, loss of pain and temperature sensation, preservation of light touch, bowel and bladder dysfunction, and possible respiratory failure. The differential diagnoses include epidural abscess and epidural hematoma. The physical exam would demonstrate loss of pain and temperature sensation, and lower extremity paresis or paralysis. Anterior spinal artery syndrome can occur during surgery and can go unnoticed while the patient is anesthetized [9].

Embolization is the preferred method to treat spinal AVMs [1, 10]. In our case, the decision was made to perform osteoplastic laminoplasties and resection of the AVM. This was because the patient had a large aneurysm that was unable to be coiled prior to surgery. Ischemia may occur during surgical resection of an AVM. Loss of blood flow to the spinal cord should be monitored by measuring spinal cord potentials [11]. Total intravenous anesthesia (TIVA) remains the standard of care as intraoperative monitoring (IOM) of SSEPs and MEPs are susceptible to amplitude changes with use of inhalational anesthetic agents and neuromuscular blockade. Hemodynamic stability and use of TIVA remains the mainstay of IOM to preserve SSEPs and MEPs [12]. For our case, SSEPs and MEPs were obtainable for upper extremities only prior to and during the surgical resection of the spinal AVM. There were no recordable evoked potentials of the lower extremities recorded prior to or during surgery.

Paraplegia is a common symptom among patients with spinal AVMs. Some patients have reversal of the paraplegia after surgical resection [1]. For our case, the dura was intentionally left open to accommodate potential spinal cord edema and swelling after her first surgery. The second feeding artery from the T9 level was embolized. Postoperative follow up angiogram was needed in our case. Our goal of anesthetic management remained as prevention of hemorrhagic complications during resection of the AVM while also preserving the spinal cord. The most recent examination by the neurosurgeon revealed spastic diplegia with loss of sensation below T10–12. It is difficult to obtain a sensory exam on the patient due to her age. Besides that, she is found to be one strong child with a fantastic attitude.

Although spinal AVMs are rare, there are cases reported in the literature of spinal AVMs in the pediatric population. However, many of them differ from our patient’s presentation. For example, one case described a 9-year-old male with a concurrent cerebral cavernous hemangioma and syringomyelia that was proposed to have a genetic etiology [13]. Another case was of a 10-year-old female that presented with an intracranial AVM with a concurrent spinal AV fistula which was proposed to be associated with hereditary disorders such as Rendu-Osler-Weber syndrome or hereditary hemorrhagic telangiectasia [14]. In both cases, the children developed symptoms at much older ages than our patient, who was 15-months old at presentation. Also, both cases are hypothesized to have a genetic etiology due to the occurrence of multiple sites of vascular abnormalities. We believe our patients’ spinal AVM was simply a congenital anomaly that is most likely not associated with her history of congenital syphilis due to the absence of any other complications of a syphilis infection.

This report highlights the importance of preparing for massive transfusion and intraoperative monitoring prior to surgical resection of spinal AVMs in the pediatric population. In patients with a history of congenital syphilis, consider how vascular changes might impact surgical approaches and treatment outcomes. More research is needed on how congenital syphilis might impact anesthetic management due to its complexity and heterogeneity of symptoms.

## Data Availability

N/A

## Abbreviations

AVM: Arteriovenous malformation
CSF: Cerebral spinal fluid
SSEPs: Somatosensory evoked potentials
MEPs: Motor-evoked potentials
IOM: Intraoperative monitoring
TIVA: Total intravenous anesthesia
